# Synthesis and Biological Evaluation of Novel 3-Alkylpyridine Marine Alkaloid Analogs with Promising Anticancer Activity

**DOI:** 10.3390/md12084361

**Published:** 2014-07-31

**Authors:** Alessandra Mirtes Marques Neves Gonçalves, Aline Brito de Lima, Maria Cristina da Silva Barbosa, Luiz Fernando de Camargos, Júlia Teixeira de Oliveira, Camila de Souza Barbosa, José Augusto Ferreira Perez Villar, André Carvalho Costa, Isabella Viana Gomes da Silva, Luciana Maria Silva, Fernando de Pilla Varotti, Fabio Vieira dos Santos, Gustavo Henrique Ribeiro Viana

**Affiliations:** 1 Núcleo de Pesquisa em Química Biológica (NQBio), Universidade Federal de São João del Rei, Campus Centro-Oeste, Av. Sebastião Gonçalves Coelho, 400, Divinópolis, MG 35.501-296, Brazil; E-Mails: alessandramirtes@gmail.com (A.M.M.N.G.); lima.alinebrito@gmail.com (A.B.L.); mariacristinaufsj@gmail.com (M.C.S.B.); luizfcamargos@yahoo.com.br (L.F.C.); julia_toliveira@hotmail.com (J.T.O.); camilasbbqi@gmail.com (C.S.B.); andrecfisio@hotmail.com (A.C.C.); isabellavianagomes@yahoo.com.br (I.V.G.S.); 2 Laboratório de Síntese Orgânica, Universidade Federal de São João del Rei, Campus Centro-Oeste, Av. Sebastião Gonçalves Coelho, 400, Divinópolis, MG 35.501-296, Brazil; E-Mail: zevillar@hotmail.com; 3 Serviço de Biologia Celular (SBC), Fundação Ezequiel Dias, Rua Conde Pereira Carneiro, 80, Belo Horizonte, MG 30.510-010, Brazil

**Keywords:** 3-alkylpyridine alkaloids, cancer, mutagenicity, apoptosis

## Abstract

Cancer continues to be one of the most important health problems worldwide, and the identification of novel drugs and treatments to address this disease is urgent. During recent years, marine organisms have proven to be a promising source of new compounds with action against tumoral cell lines. Here, we describe the synthesis and anticancer activity of eight new 3-alkylpyridine alkaloid (3-APA) analogs in four steps and with good yields. The key step for the synthesis of these compounds is a Williamson etherification under phase-transfer conditions. We investigated the influence of the length of the alkyl chain attached to position 3 of the pyridine ring on the cytotoxicity of these compounds. Biological assays demonstrated that compounds with an alkyl chain of ten carbon atoms (**4c** and **5c**) were the most active against two tumoral cell lines: RKO-AS-45-1 and HeLa. Micronucleus and TUNEL assays showed that both compounds are mutagenic and induce apoptosis. In addition, Compound **5c** altered the cellular actin cytoskeleton in RKO-AS-45-1 cells. The results suggest that Compounds **4c** and **5c** may be novel prototype anticancer agents.

## 1. Introduction

Cancer remains one of the leading causes of death in the world, particularly in developing countries [1]. In this context, the identification of novel drugs and treatments to address this situation is urgent [2]. The discovery of secondary metabolites from marine organisms has provided a potential source of new compounds with action against tumor cell lines [3].

**Figure 1:**
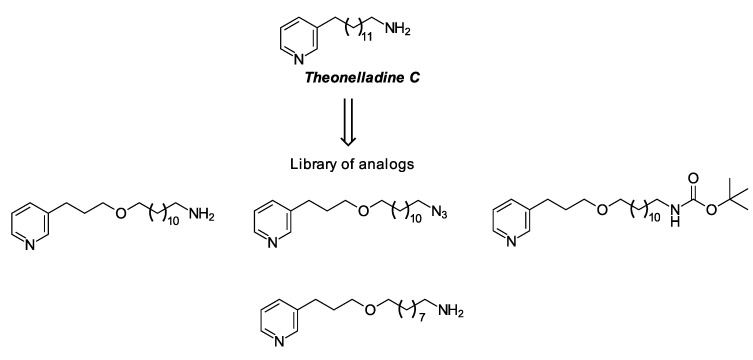
Theonelladine C and some examples of its synthetic analogs.

Marine sponges are a rich source of secondary metabolites [4]. These metabolites have been widely reported, as well as many synthetic analogs with interesting antitumor activity [5,6,7,8,9,10]. The marine sponge, *Theonella* sp. (order Lithistida), is a rich source of structurally-diverse, biologically-active peptides, such as cyclopeptide perthamides G–K [11], glycopeptide theonellamides A–G [12] and 3-alkylpyridine alkaloids (3-APAs), including theonelladines A–D [13]. Although different classes of metabolites have been isolated from this group of marine sponges, these compounds are typically highlighted for their remarkable cytotoxic activity against tumor human cell lines [11,12], as are their analogs [14]. A small series of 3-APA analogs of theonelladine C (Figure 1) recently synthesized by our research group exhibited antiprotozoal activity [15,16] and pro-apoptotic action against a human colon carcinoma (RKO-AS-45-1) cell line [14]. We also observed that their cytotoxicity appeared to be affected by the alkyl chain length (9 or 12 carbon atoms) and the type of functional group attached to the end of this chain (e.g., azide or amine) (Figure 1).

Apoptosis is a crucial process in cellular physiology [17], and this cell death process can be triggered by the action of anticancer agents [18]. However, the apoptotic process is not associated with the activation of immune response, a desirable feature in cancer treatment [19].

The mitochondrial or intrinsic apoptotic pathway is activated in response to cellular stress signals that can originate from DNA damage, failures in the cell cycle and the loss of cell survival factors [20]. Different methodologies are employed to identify agents able to induce DNA damage, and the micronucleus assay is an important alternative. Micronuclei (MNs) can be induced following exposure to aneugenic and clastogenic agents, and their quantification provides a sensitive tool for the assessment of genotoxicity [21]. Moreover, studies have shown that micronucleated cells can be eliminated by apoptosis [22] and that caspases-9, -8, and -3 are involved in this process [23]. Thus, the identification of a compound able to induce MN formation permits the inference of its potential to activate apoptotic processes, providing relevant information about its potential as an anticancer candidate.

In this study, classical methodologies were applied to assess cell viability, the induction of chromosomal alterations and the triggering of the apoptotic process in different cell lines after treatment with some synthetic alkaloids to evidence the molecular mode of action of a new generation of 3-APA analogs.

## 2. Results and Discussion

### 2.1. Chemistry

#### Synthesis

Based on our previous results [14], we designed a new series of 3-APA analogs with a long alkyl chain length ranging from six to 12 carbon atoms to verify the dependency of the cytotoxic response upon the alkyl chain length. A hydroxyl group was attached to the end of this chain on Compounds **5a**–**d** to improve their water solubility. The aqueous solubility favors the ease of manipulation and biological assays in the laboratory, as well as the formulation development of new chemical entities [24,25]. In addition, the presence of a hydroxyl group would favor future structural modifications by functional group interconversion.

**Scheme 1:**
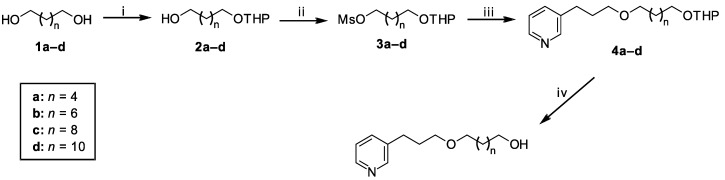
Synthesis of 3-alkylpyridine alkaloid (3-APA) analogs **4a**–**d** and **5a**–**d**.

The synthesis of the new 3-APA analogs is depicted in Scheme 1. The key step for the synthesis of these compounds was a Williamson etherification under phase-transfer catalysis (PTC), which favors the preparation of ethers under mild conditions. 1,*n*-Diols with six, eight, 10 or 12 carbon atoms, **1a**–**d**, were selectively protected to generate the corresponding monotetrahydropyranyl acetals, **2a**–**d**, with 74%–89% yields, in a reaction catalyzed by aqueous NaHSO_4_ in contact with a 3,4-dihydro-2*H*-pyran (DHP) hexane layer [26]. The mono-protected alcohols, **2a**–**d**, were easily mesylated under classical conditions to give intermediates **3a**–**d**. The etherification of commercial 3-(pyrid-3-yl)propan-1-ol with mesylated derivatives **3a**–**d** using a phase-transfer catalyst produced the protected Compounds **4a**–**d** in good yields. Deprotection with HCl [27] afforded Compounds **5a**–**d** in 71%–100% yields.

Reagents, conditions, and yields: (i) NaHSO_4_, DHP, DMSO, hexane, 40 °C, 16 h, 74%–89%; (ii) MsCl, Et_3_N, CH_2_Cl_2_, RT, 10 h, 77%–87%; (iii) 3-(pyrid-3-yl)propan-1-ol, NaOH/H_2_O, Bu_4_N^+^Br^−^, Et_2_O, RT, 72 h, 57%–73%; (iv) MeOH, HCl, RT, 12 h, 71%–100%.

### 2.2. Biological Evaluation

#### 2.2.1. Cell Viability

**Figure 2:**
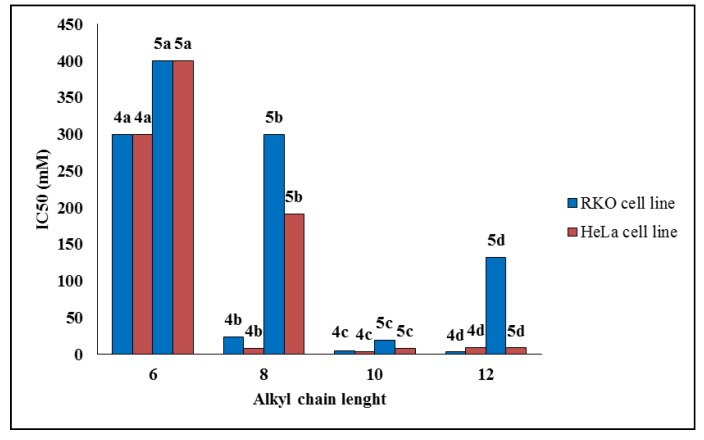
Correlation between IC_50_ and the alkyl chain length of Compounds **4a**–**d** and **5a**–**d** against RKO-AS-45-1 and HeLa cell lines.

All synthesized compounds were evaluated* in vitro* for their anticancer potential against two human cancer cell lines (colon carcinoma RKO-AS-45-1 and uterine carcinoma HeLa). The compounds were also tested in a non-cancerous human cell line (lung fibroblast WI-26VA4) to determine the selective index (SI). A colorimetric MTT assay showed that Compounds **4c**, **4d** and **5c** presented antitumor activity* in vitro* (Table 1), showing IC_50_ values of 5.1, 3.2 and 19.1 μM, respectively, against RKO-AS-45-1 cells. These same compounds presented IC_50_ values ranging from 4.0 to 9.4 μM against the HeLa cell line. Etoposide, an anticancer agent that is used as a standard drug, was tested in parallel [28]. Based on these data, it was possible to establish a relationship between IC_50_ values and the alkyl chain length of the compounds. As observed in Figure 2, the set of compounds with ten carbon atoms showed the lowest set of IC_50_ values against the two tumoral cell lines. Furthermore, Compound **5c** exhibited the highest SI of the series, 5.18 and 11.65 for the RKO-AS-45-1 and HeLa cells, respectively. Therefore, Compounds **4c** (one of the most active compounds) and **5c** were selected for further study of their mode of action.

**Table 1:** *In vitro* cytotoxic activity of new 3-APA analogs **4a**–**d** and **5a**–**d** against RKO-AS-45-1 and HeLa cell lines.

Compound	IC_50_ (μM) ± SD *			Selectivity Index (SI)	
	RKO AS-45-1	HeLa	WI-26VA4	RKO AS-45-1	HeLa
**4a**	>300	>300	>300	ND **	ND
**4b**	23.7 ± 1.7	8.1 ± 2.7	37.8 ± 5.0	1.59	4.66
**4c**	5.1 ± 1.1	4.0 ± 0.8	6.4 ± 0.7	1.25	1.60
**4d**	3.2 ± 1.7	9.4 ± 0.7	11.3 ± 1.4	3.53	1.20
**5a**	>400	>400	>400	ND	ND
**5b**	>300	191.8 ± 10.1	167.0 ± 10.5	ND	0.87
**5c**	19.1 ± 4.4	8.5 ± 2.4	99.1 ± 11.2	5.18	11.65
**5d**	131.9 ± 16.8	8.8 ± 1.9	34.1 ± 6.5	0.25	3.87
**Etoposide**	1.4 ± 0.6	2.7 ± 0.4	ND	-	-

* Values are the average ± standard deviation; ** ND: not determined.

#### 2.2.2. Mutagenicity Assay

**Figure 3:**
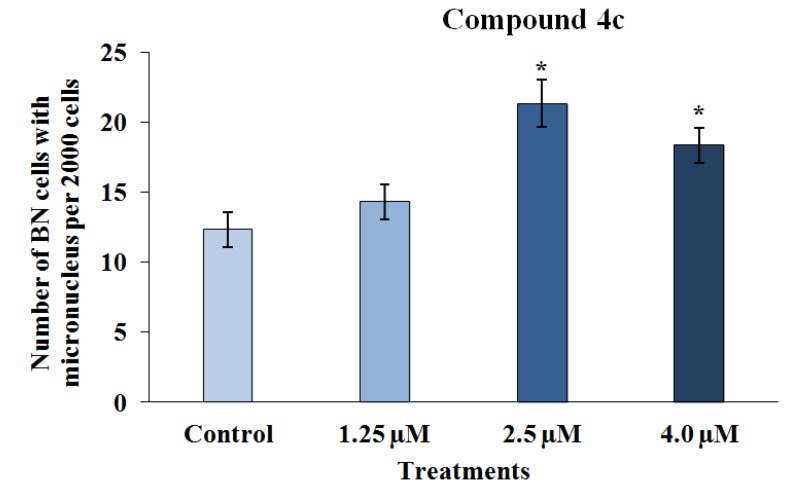
The results of the cytokinesis-block micronucleus assay performed with RKO-AS-45-1 cells treated with different concentrations of 3-APA synthetic derivative **4c**. As observed, the micronucleus frequency in 2000 binucleated cells treated with the two highest concentrations of the **4c** compound was statistically higher than that identified in the control group, indicating that the 3-APA synthetic derivative was mutagenic at these concentrations. * Statistically different of the Control (*p* < 0.05)

**Figure 4:**
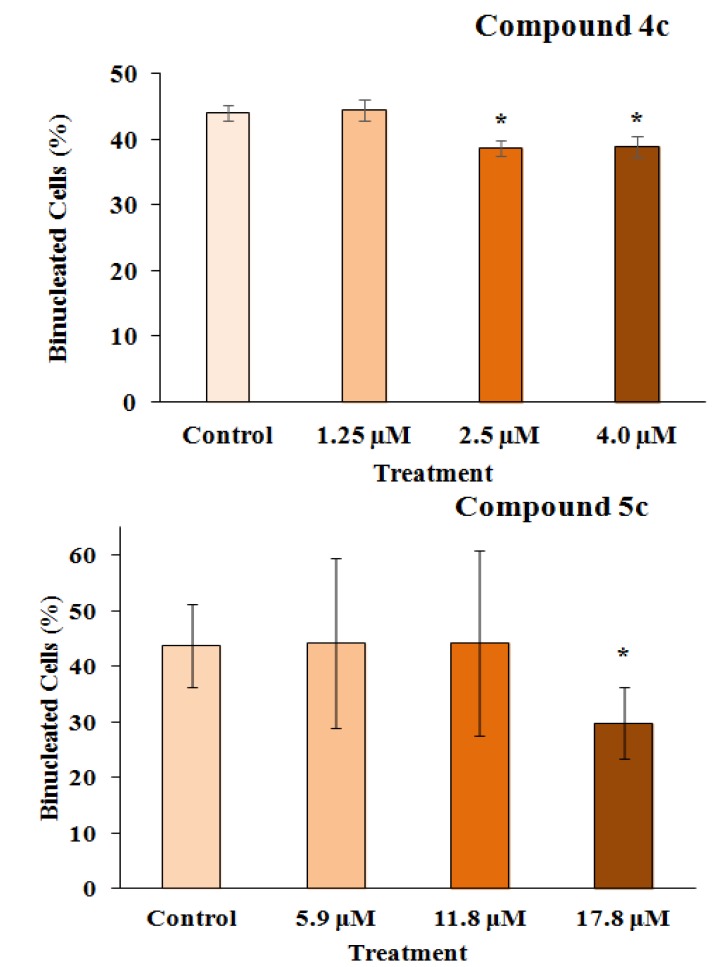
The frequency of binucleation (%) in 300 cells counted after treating the RKO-AS-45-1 cell line with different concentrations of Compounds **4c** and **5c**. As observed, Compound **5c** induced a reduction in the proliferation rate of the treated cells at the highest concentration assessed, with the percentage of binucleated cells being lower than that observed in the control group. * Statistically different of the Control (*p* < 0.05).

Genotoxicity describes many different DNA endpoints, including DNA adduct formation, point mutation, chromosome breakage and chromosome copy number changes [29]. These processes are more highly scrutinized in pharmaceuticals than in any other chemical sector, because sustained and often high systemic drug exposure is generally required for therapeutic efficacy [30]. Moreover, the anticancer activities of chemotherapeutic drugs (such as alkylating agents and platinum compounds) and radiotherapy is to a large extent directly related to the ability to induce DNA damage [31]. In this way, the genotoxicity of the two 3-APA analog derivatives, **4c** and **5c**, was assessed by employing the micronucleus mutagenicity assay *in vitro* using the RKO-AS-45-1 cell line as a biological model. The results presented in Figure 3 show that the MN frequency observed after the exposure of the cells to Compound **4c** (2.5 and 4.0 μM) was significantly higher than that detected in the control. This result indicates that this synthetic alkaloid analog was able to cause DNA breaks and/or aneuploidy under the conditions employed. The lower concentrations evaluated did not induce significant alterations in the MN frequency. Therefore, the two highest concentrations assessed reduced the binucleation frequency when compared with the negative control (Figure 4). These results indicate that this compound, at concentrations near the IC_50_, reduced the proliferation rate, most likely due to the DNA damage observed in the MN assay.

**Figure 5:**
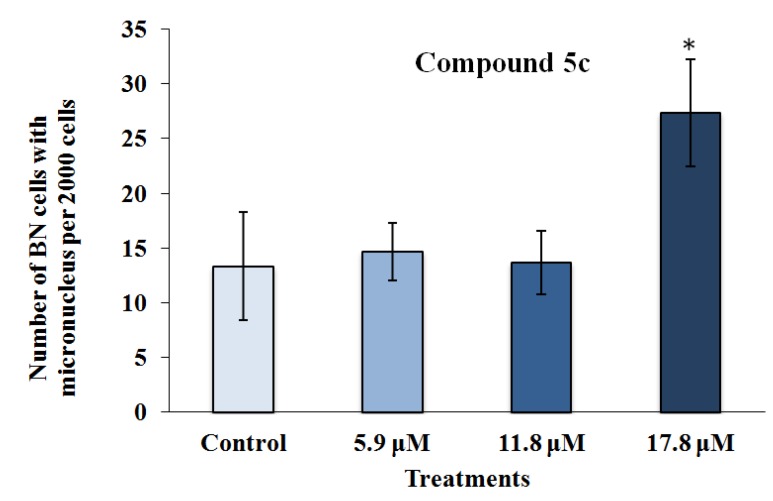
The results of the cytokinesis-block micronucleus assay performed with RKO-AS-45-1 cells treated with different concentrations of 3-APA synthetic derivative **5c**. As observed, the micronucleus frequency in 2000 binucleated cells treated with the highest concentration of the **5c** compound was statistically higher than that identified in the control group, indicating that the 3-APA synthetic derivative was mutagenic at this concentration. * Statistically different of the Control (*p* < 0.05)*.*

As observed in Figure 5, the highest concentration assessed (17.8 μM) of Compound **5c** induced a number of micronucleated cells, which was significantly higher than that found in the control. Complementarily, the analysis of the frequency of binucleation (Figure 4) in the RKO-AS-45-1 cell line showed that the highest analyzed concentration of this compound reduced the proliferation rate of these cells.

RKO-AS-45-1 is a human tumoral cell line that expresses p53, a protein that is responsible for cell cycle arrest at the G1/S checkpoint in response to DNA damage. According to Brusehafer* et al.* (2014) [32],* in vitro* model systems for genotoxicity testing are sometimes prone to misleading positive results, and some of this loss of predictive power may be caused by p53 inactivation in some cell models. Within this context, when choosing cell lines for* in vitro* genotoxicity assays, it is important to be aware of the p53 status and to know how this feature will influence genotoxic and cytotoxic responses to the various types of tested chemicals [33]. The results observed in the MN assay and in the binucleation frequency analysis are in agreement, because the DNA damage detected in the MN assay most likely resulted in cell cycle arrest. According to Amundson (1998) [34], wild-type p53 cells can clearly exhibit both of the major cellular responses to DNA damage: cell cycle arrest and apoptosis. In addition, the activation of p53 can lead to cell cycle arrest to prevent the proliferation of damaged cells and to allow DNA repair prior to replication and mitosis or to induce apoptosis to eliminate irreparably damaged cells. Moreover, according to Utani* et al.* (2010) [35], the induction of MNs by chemical agents can be related to the induction of apoptosis.

#### 2.2.3. TUNEL Assay

**Figure 6:**
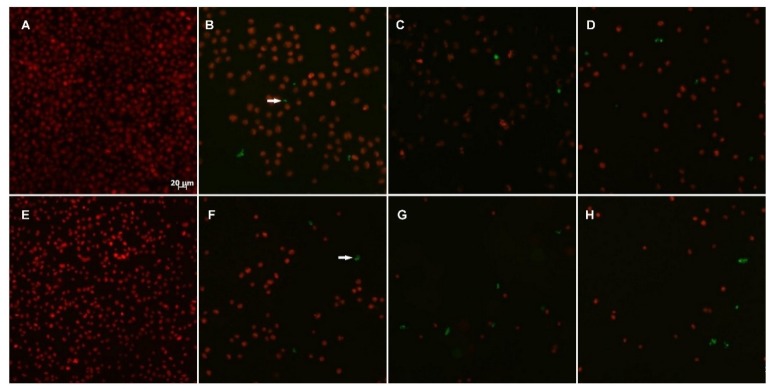
Apoptosis in HeLa (**A**–**D**) and RKO-AS-45-1 (**E**–**H**) cells. Cells were incubated (48 h) with Compounds **4c** and **5c** at different concentrations: non-treated cells (**A** and **E**), etoposide at 1 μM (**B** and **F**); **5c** at 8.5 μM (**C**); **4c** at 19.1 μM (**D**), **5c** at 19.1 μM (**G**), and **4c** at 5.1 μM (**H**). The arrow indicates cell death by apoptosis (green). The total cellular DNA content is stained in red. Scale bar: 20 μm.

The induction of apoptosis is considered an efficient strategy for the identification of potential antitumor drugs [36]. To investigate the possible apoptosis-inducing action of Compounds **4c** and **5c**, specific DNA fragments in RKO-AS-45-1 and HeLa cells were detected by a TUNEL assay in parallel with etoposide, a known apoptosis-inducing drug [37]. The results presented in Figure 6 indicate that Compounds **4c** and **5c** promoted apoptosis in RKO-AS-45-1 and HeLa cells, reducing the number of viable cells. The apoptotic behavior of isolated or synthetic alkaloids is associated with mitochondrial membrane depolarization [38], phosphatidylserine externalization [39] and alteration of the patterns of cytoskeleton polymerization [40].

#### 2.2.4. Cytoskeleton Assembly

**Figure 7:**
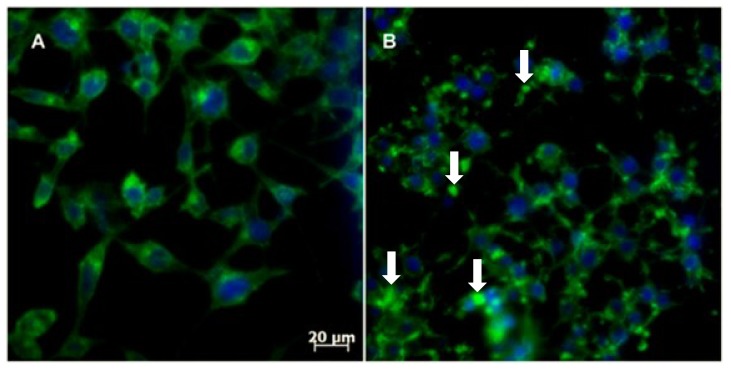
The effect of 3-APA **5c** on the actin cytoskeleton of RKO-AS-45-1 cells: (**A**) non-treated cells; (**B**) **5c** at 19.1 μM. The arrows indicate that the treatment caused an accentuated alteration of the cellular actin network (green fluorescence).

Reorganization of the cytoskeletal network is crucial for mitosis and is important due to centrosome reorientation until the division of the daughter cells [41]. Actin dynamics are involved in a crucial step of cell division: the formation of the contractile ring [42]. Within this context, we assayed 3-APA analog **5c** (the most selective compound of the series) in RKO-AS-45-1 cells to provide evidence of changes in cytoskeleton assembly. The results showed that actin polymerization is altered after treatment with Compound **5c** (Figure 7). Similar results have already been reported for another class of marine compounds, the swinholide-type macrolides [43].

Thus, the action of a compound that can alter the actin dynamics may be sufficient to block mitosis. Indeed, cytotoxic compounds with mitosis-blocking action, such as taxanes, are used as chemotherapy agents [44].

## 3. Experimental Section

### 3.1. Chemistry

Reagents and solvents were purchased as reagent grade and used without further purification. NMR spectra were recorded using Bruker Avance DRX-200 or DRX-400 spectrometers. Chemical shifts are reported as *δ* (ppm) downfield from tetramethylsilane (TMS), and the *J* values are reported in Hz. IR spectra were recorded using a Shimadzu IRAffinity-1 Fourier transform spectrometer. Low-resolution mass spectra (LRMS) were recorded using an ESI Bruker Daltonics amaZon SL Ion Trap mass spectrometer, and high-resolution mass spectrometry (HRMS) was performed using an ESI micrOTOF-QII Bruker mass spectrometer. Column chromatography was performed with silica gel 60, 70–230 mesh (Merck, Darmstadt, Germany).

### 3.2. Synthesis

General procedure for the synthesis of monotetrahydropyranyl acetals **2a**–**d**: A mixture of 1,*n*-diols **1a**–**d** (1.0 mmol), DHP-hexane 3% (*v*/*v*) (1.96 mmol) and aqueous 5 M NaHSO_4_ (01 mL) was prepared. To 1,10-decanediol **1c** and 1,12-dodecanediol **1d**, 0.2 mL of DMSO were also added to the mixture at the beginning of the reaction. This mixture was stirred at 40 °C for 16 h and then extracted with hexane (3 × 20 mL). The combined organic phases were dried (Na_2_SO_4_), filtered and then evaporated under reduced pressure. The residue obtained was chromatographed using silica gel using hexane/EtOAc (8:2) to yield pure Compounds **2a**–**d**.

6-(oxan-2-yloxy)hexan-1-ol **2a**: Yield 89%, colorless oily product: IR (KBr): 𝑣 = 3398, 2939, 2865, 1406, 1440, 1138, 1120, 857 cm^−1^. ^1^H NMR (CDCl_3_, 400 MHz): δ 1.33–1.42 (m, 4H), 1.50–1.61 (m, 8H), 1.69–1.75 (m, 1H), 1.79–1.87 (m, 1H,), 3.39 (dt, 1H, *J* = 9.6 Hz, *J* = 6.5 Hz), 3.48–3.53 (m, 1H), 3.64 (t, 2H, *J* = 6.6 Hz), 3.74 (dt, 1H, *J* = 9,6 Hz, *J* = 6.8 Hz), 3.84–3.90 (m, 1H), 4.57 (t, 1H, *J* = 2.6 Hz) ppm. ^13^C NMR (CDCl_3_, 100 MHz): δ 19.69, 25.45, 25.52, 26.00, 29.65, 30.75, 32.66, 62.85, 67.49, 98.88 ppm. LRMS (ESI) *m*/*z* 225.0 [M + Na]^+^.

8-(oxan-2-yloxy)octan-1-ol **2b**: Yield 74%, colorless oily product: IR (KBr): 𝑣 = 3425, 2921, 2856, 1382, 1440, 1128, 1120, 857 cm^−1^. ^1^H NMR (CDCl_3_, 400 MHz): δ 1.28–1.36 (m, 8H), 1.50–1.61 (m, 8H), 1.69–1.75 (m, 1H), 1.80–1.86 (m, 1H), 3.37 (dt, 1H, *J* = 9.6 Hz, *J* = 6.7 Hz), 3.47–3.52 (m, 1H), 3.63 (t, 2H, *J* = 6.6 Hz), 3.73 (dt, 1H, *J* = 9,6 Hz, *J* = 6.9 Hz), 3.84–3.89 (m, 1H), 4.57 (t, 1H, *J* = 2.6 Hz) ppm. ^13^C NMR (CDCl_3_, 100 MHz): δ 19.63, 25.43, 25.63, 26.10, 29.30, 29.37, 29.65, 30.72, 32.70, 62.30, 62.91, 67.61, 98.80 ppm. LRMS (ESI) *m*/*z* 253.0 [M + Na]^+^.

10-(oxan-2-yloxy)decan-1-ol **2c**: Yield 89%, colorless oily product: IR (KBr): 𝑣 = 3305, 2926, 2854, 1400, 1440, 1128, 1120, 857 cm^−1^. ^1^H NMR (CDCl_3_, 400 MHz): δ 1.29–1.30 (m, 12H), 1.50–1.62 (m, 8H), 1.69–1.76 (m, 1H), 1.81–1.86 (m, 1H), 3.39 (dt, 1H, *J* = 9.6 Hz, *J* = 6.7 Hz), 3.48–3.54 (m, 1H), 3.64 (t, 2H, *J* = 6.6 Hz), 3.73 (dt, 1H, *J* = 9,6 Hz, *J* = 6.9 Hz), 3.85–3.91 (m, 1H), 4.59 (t, 1H, *J* = 2.6 Hz) ppm. ^13^C NMR (CDCl_3_, 100 MHz): δ 19.65, 25.46, 25.69, 26.18, 29.36, 29.41, 29.46, 29.49, 29.69, 30.74, 32.75, 62.31, 62.98, 67.65, 98.80 ppm. LRMS (ESI) *m*/*z* 281.0 [M + Na]^+^.

12-(oxan-2-yloxy)dodecan-1-ol **2d**: Yield 74%, colorless oily product: IR (KBr): 𝑣 = 3429, 2926, 2855, 1420, 1456, 1124, 1122, 869 cm^−1^. ^1^H NMR (CDCl_3_, 400 MHz): δ 1.21–1.30 (m, 16H), 1.44–1.55 (m, 8H), 1.61–1.69 (m, 1H), 1.73–1.81 (m, 1H), 3.32 (dt, 1H, *J* = 9.6 Hz, *J* = 6.7 Hz), 3.41–3.47 (m, 1H), 3.57 (t, 2H, *J* = 6.6 Hz), 3.67 (dt, 1H, *J* = 9,6 Hz, *J* = 6.9 Hz), 3.78–3.84 (m, 1H), 4.52 (t, 1H, *J* = 2.6 Hz) ppm. ^13^C NMR (CDCl_3_, 100 MHz): δ 19.67, 25.47, 25.71, 26.20, 29.39, 29.44, 29.53, 29.71, 30.75, 32.77, 62.32, 63.03, 67.68, 98.81 ppm. LRMS (ESI) *m*/*z* 308.9 [M + Na]^+^.

General procedure for the synthesis of mesylate compounds **3a**–**d**: A solution of monotetrahydropyranyl acetals 2**a**–**d** (9.9 mmol) in CH_2_Cl_2_ (50 mL) was cooled to 0 °C. Et_3_N (39.6 mmol) and methanesulfonyl chloride (19.8 mmol) was added. The reaction mixture was stirred for 10 h and then allowed to warm to room temperature. The reaction mixture was poured into crushed ice (50 mL) and then extracted with methylene chloride (3 × 30 mL). The organic layer was dried (Na_2_SO_4_), filtered and evaporated under reduced pressure. The residue obtained was purified by column chromatography (SiO_2_, hexane/EtOAc 1:1) to yield pure mesylated Compounds **3a**–**d**.

6-(oxan-2-yloxy)hexyl methanesulfonate **3a**: Yield 87%, yellow oily product: IR (KBr): 𝑣 = 2929, 2865, 1420, 1354, 1174, 1124, 1122, 856 cm^−1^. ^1^H NMR (CDCl_3_, 400 MHz): δ 1.37–1.44 (m, 4H), 1.52–1.60 (m, 8H), 1.72–1.79 (m, 1H), 1.80–1.86 (m, 1H), 3.01 (s, 3H), 3.39 (dt, 1H, *J* = 9.6 Hz, *J* = 6.4 Hz), 3.48–3.53 (m, 1H), 3.74 (dt, 1H, *J* = 9,6 Hz, *J* = 6.7 Hz), 3.84–3.89 (m, 1H), 4.23 (t, 1H, *J* = 6.5 Hz), 4.57 (t, 1H, *J* = 2.6 Hz) ppm. ^13^C NMR (CDCl_3_, 100 MHz): δ 19.71, 25.27, 25.44, 25.72, 29.05, 29.50, 30.74, 37.34, 62.44, 67.32, 70.01, 98.92 ppm. LRMS (ESI) *m*/*z* 303.0 [M + Na]^+^.

8-(oxan-2-yloxy)octyl methanesulfonate **3b**: Yield 79%, yellow oily product: IR (KBr): 𝑣 = 2937, 2858, 1400, 1354, 1174, 1131, 1120, 857 cm^−1^. ^1^H NMR (CDCl_3_, 400 MHz): δ 1.34–1.41 (m, 8H), 1.52–1.59 (m, 8H), 1.71–1.77 (m, 1H), 1.80–1.85 (m, 1H), 3.00 (s, 3H), 3.38 (dt, 1H, *J* = 9.6 Hz, *J* = 6.6 Hz), 3.48–3.53 (m, 1H), 3.73 (dt, 1H, *J* = 9,6 Hz, *J* = 6.8 Hz), 3.84–3.90 (m, 1H), 4.22 (t, 1H, *J* = 6.6 Hz), 4.58(t, 1H, *J* = 2.7 Hz) ppm. ^13^C NMR (CDCl_3_, 100 MHz): δ 19.67, 25.32, 28.91, 29.05, 29.12, 29.20, 29.33, 29.37, 29.42, 37.31, 62.36, 67.54, 70.12, 98.85 ppm. LRMS (ESI) *m*/*z* 330.9 [M + Na]^+^.

10-(oxan-2-yloxy)decyl methanesulfonate **3c**: Yield 77%, yellow oily product: IR (KBr): 𝑣 = 2924, 2854, 1410, 1354, 1176, 1138, 1122, 857 cm^−1^. ^1^H NMR (CDCl_3_, 400 MHz): δ 1.29–1.40 (m, 12H), 1.52–1.61 (m, 8H), 1.71–1.76 (m, 1H), 1.80–1.85 (m, 1H), 3.01 (s, 3H), 3.38 (dt, 1H, *J* = 9.6 Hz, *J* = 6.7 Hz), 3.48–3.53 (m, 1H), 3.73 (dt, 1H, *J* = 9,6 Hz, *J* = 6.9 Hz), 3.85–3.89 (1H, m), 4.22 (t, 1H, *J* = 6.6 Hz), 4.58 (t, 1H, *J* = 2.6 Hz) ppm. ^13^C NMR (CDCl_3_, 100 MHz): δ 19.70, 25.39, 25.48, 26.19, 28.98, 29.10, 29.33, 29.40, 29.72, 30.78, 37.35, 62.36, 67.65, 70.16, 98.85 ppm. LRMS (ESI) *m*/*z* 358.9 [M + Na]^+^.

12-(oxan-2-yloxy)dodecyl methanesulfonate **3d**: Yield 80%, yellow oily product: IR (KBr): 𝑣 = 2922, 2852, 1400, 1354, 1176, 1136, 1122, 857 cm^−1^. ^1^H NMR (CDCl_3_, 400 MHz): δ 1.26–1.39 (m, 16H), 1.51–1.62 (m, 8H), 1.71–1.76 (m, 1H), 1.81–1.85 (m, 1H), 3.00 (s, 3H), 3.38 (dt, 1H, *J* = 9.6 Hz, *J* = 6.7 Hz), 3.47–3.52 (m, 1H), 3.72 (dt, 1H, *J* = 9,6 Hz, *J* = 6.9 Hz), 3.84–3.90 (m, 1H), 4.22 (t, 1H, *J* = 6.6 Hz), 4.57 (t, 1H, *J* = 2.6 Hz) ppm. ^13^C NMR (CDCl_3_, 100 MHz): δ 19.74, 25.44, 26.25, 29.05, 29.14, 29.42, 29.50, 29.56, 30.77, 37.38, 62.39, 67.72, 70.23, 98.88 ppm. LRMS (ESI) *m*/*z* 386.9 [M + Na]^+^.

General procedure for the synthesis of **4a**–**d**: A mixture of 3-(pyrid-3-yl)propan-1-ol (3.42 mmol), each mesylate compound (**3a**–**d**; 3.42 mmol) and tetrabutylammonium bromide (1.13 mmol) was prepared in 10 mL of Et_2_O with 5 mL of aqueous sodium hydroxide (50% *w*/*v*). Each mixture was stirred vigorous at room temperature for 72 h and then extracted with Et_2_O (3 × 30 mL). The combined organic phases were dried (Na_2_SO_4_), filtered and evaporated under reduced pressure. The residue obtained was chromatographed on silica gel using hexane/EtOAc (1:1) to yield pure Compounds **4a**–**d**.

1-[3-(pyridin-3-yl)propoxy]-6-(oxan-2-yloxy)hexane **4a**: Yield 69%, yellow oily product: IR (KBr): 𝑣 = 2929, 2865, 1456, 1421, 1124, 1120, 983, 857 cm^−1^. ^1^H NMR (CDCl_3_, 400 MHz): δ 1.30–1.43 (m, 8H), 1.50–1.63 (m, 6H), 1.66–1.75 (m, 1H), 1.79–1.92 (m, 1H), 2.70 (t, 2H, *J* = 7.4 Hz), 3.38–3.43 (m, 5H), 3.47–3.53 (m, 1H), 3.74 (dt, 1H, *J* = 9.6 Hz, *J* = 6.8 Hz), 3.84–3.90 (m, 1H), 4.58 (t, 1H, *J* = 2.6 Hz), 7.19–7.23 (m, 1H), 7.52 (d, 1H, *J* = 7.8 Hz), 8.43–8.46 (m, 2H) ppm. ^13^C NMR (CDCl_3_, 100 MHz): δ 19.67, 25.45, 26.09, 29.48, 29.67, 30.74, 30.97, 62.33, 67.53, 69.40, 70.94, 98.83, 123.22, 135.84, 137.19, 147.27, 149.97 ppm. HRMS [M + Na]^+^ = 344.2193 (theoretical 344.2202).

1-[3-(pyridin-3-yl)propoxy]-8-(oxan-2-yloxy)octane **4b**: Yield 57%, yellow oily product: IR (KBr): 𝑣 = 2931, 2856, 1454, 1421, 1120, 1118, 987, 857 cm^−1^. ^1^H NMR (CDCl_3_, 400 MHz): δ 1.29–1.42 (m, 12H), 1.50–1.64 (m, 6H), 1.70–1.76 (m, 1H), 1.82–1.94 (m, 1H), 2.72 (t, 2H, *J* = 7.9 Hz), 3.37–3.44 (m, 5H), 3.47–3.55 (m, 1H), 3.74 (dt, 1H, *J* = 9.6 Hz, *J* = 6.9 Hz), 3.86–3.91 (m, 1H), 4.59 (t, 1H, *J* = 2.6 Hz), 7.21–7.24(m, 1H), 7.53 (d, 1H, *J* = 7.8 Hz), 8.44–8.48 (m, 2H) ppm. ^13^C NMR (CDCl_3_, 100 MHz): δ 19.65, 25.44, 26.13, 29.37, 29.45, 29.67, 30.72, 30.94, 62.30, 67.60, 69.37, 71.01, 98.80, 123.21, 135.85, 137.19, 147.21, 149.92 ppm. HRMS [M + Na]^+^ = 372.2508 (theoretical 372.2515).

1-[3-(pyridin-3-yl)propoxy]-10-(oxan-2-yloxy)decane **4c**: Yield 73%, yellow oily product: IR (KBr): 𝑣 = 2926, 2852, 1463, 1440, 1136, 1122, 985, 857 cm^−1^. ^1^H NMR (CDCl_3_, 400 MHz): δ 1.30–1.36 (m, 16H), 1.50–1.64 (m, 6H), 1.69–1.76(m, 1H), 1.81–1.93 (m, 1H), 2.71 (t, 2H, *J* = 7.9 Hz), 3.36–3.43 (m, 5H), 3.47–3.54 (m, 1H), 3.73 (dt, 1H, *J* = 9.6 Hz, *J* = 6.9 Hz), 3.85–3.91 (m, 1H), 4.58 (t, 1H, *J* = 2.6 Hz), 7.20–7.23 (m, 1H), 7.51 (d, 1H, *J* = 8.0 Hz), 8.44–8.47 (m, 2H) ppm. ^13^C NMR (CDCl_3_, 100 MHz): δ 19.68, 25.48, 26.20, 29.45, 29.50, 29.72, 30.76, 30.98, 62.31, 67.65, 69.39, 71.06, 98.80, 123.20, 135.82, 137.20, 147.28, 150.00 ppm. LRMS (ESI) *m*/*z* 378.2 [M + H]^+^. HRMS [M + Na]^+^ = 400.2817 (theoretical 400.2828).

1-[3-(pyridin-3-yl)propoxy]-12-(oxan-2-yloxy)dodecane **4d**: Yield 66%, yellow oily product: IR (KBr): 𝑣 = 2926, 2854, 1456, 1440, 1130, 1122, 983, 857 cm^−1^. ^1^H NMR (CDCl_3_, 400 MHz): δ 1.27–1.31 (m, 20H), 1.54–1.63 (m, 6H), 1.69–1.75 (m, 1H), 1.79–1.93 (m, 1H), 2.71 (t, 2H, *J* = 7.9 Hz), 3.35–3.43 (m, 5H), 3.47–3.53 (m, 1H), 3.72 (dt, 1H, *J* = 9.5 Hz, *J* = 6.9 Hz), 3.85–3.90 (m, 1H), 4.58 (t, 1H, *J* = 2.6 Hz), 7.19–7.22 (m, 1H), 7.51 (d, 1H, *J* = 7,8 Hz), 8.44–8.46 (m, 2H) ppm. ^13^C NMR (CDCl_3_, 100 MHz): δ 19.74, 25.44, 26.22, 29.49, 29.57, 29.74, 30.78, 30.99, 62.32, 67.67, 69.41, 71.09, 98.82, 123.23, 135.84, 137.23, 147.30, 150.02 ppm.

General procedure for the synthesis of **5a**–**d**: To a solution of each compound (**4a**–**d**; 2.60 mmol) in MeOH (50 mL) was added 5.2 mL of 1 M HCl. After the addition, the reaction was stirred for 12 h at room temperature, and the solution was concentrated to 1/4 of its volume. The pH was brought to 10 by the addition of 2 M NaOH, and the solution was then extracted with EtOAc (3 × 30 mL). The combined organic phases were dried (Na_2_SO_4_), filtered and evaporated under reduced pressure. The residue obtained was chromatographed on silica gel using EtOAc to yield pure Compounds **5a**–**d**.

6-[3-(pyridin-3-yl)propoxy]hexan-1-ol **5a**: Yield 92%, yellow oily product: IR (KBr): 𝑣 = 3352, 2933, 2858, 1456, 1423, 1357, 1130, 1114, 980, 830 cm^−1^. ^1^H NMR (CDCl_3_, 400 MHz): δ 1.23–1.42 (m, 4H), 1.56–1.63 (m, 4H), 1.89 (m, 2H), 2.71 (t, 2H, *J* = 7.38Hz), 3.38–3.42 (m, 4H), 3.65 (t, 2H, *J* = 6.5 Hz), 7.20–7.24 (m, 1H), 7.52 (d, 1H, *J* = 7,8 Hz), 8.42–8.45 (m, 2H) ppm. ^13^C NMR (CDCl_3_, 100 MHz): δ 25.53, 25.95, 29.40, 29.61, 30.85, 32.70, 62.66, 69.28, 70.80, 123.30, 135.97, 137.22, 147.12, 149.86 ppm. HRMS [M + H]^+^ = 238.1801 (theoretical 238.1807).

8-[3-(pyridin-3-yl)propoxy]octan-1-ol **5b**: Yield 100%, yellow oily product: IR (KBr): 𝑣 = 3350, 2927, 2854, 1456, 1423, 1357, 1130, 1114, 985, 856 cm^−1^. ^1^H NMR (CDCl_3_, 400 MHz): δ 1.25–1.40 (m, 8H), 1.54–1.59 (m, 4H), 1.89 (m, 2H), 2.71 (t, 2H, *J* = 7.8 Hz), 3.38–3.42 (m, 4H), 3.64 (t, 2H, *J* = 6.6 Hz), 7.20–7.24 (m, 1H), 7.52 (d, 1H, *J* = 7.8 Hz), 8.41–8.45 (m, 2H) ppm. ^13^C NMR (CDCl_3_, 100 MHz): δ 25.64, 26.04, 29.29, 29.33, 29.39, 29.63, 30.86, 32.72, 62.73, 69.27, 70.93, 123.30, 136.01, 137.27, 147.06, 149.76 ppm. HRMS [M + H]^+^ = 266.2117 (theoretical 266.2120).

10-[3-(pyridin-3-yl)propoxy]decan-1-ol **5c**: Yield 71%, yellow oily product: IR (KBr): 𝑣 = 3429, 2926, 2854, 1456, 1433, 1352, 1134, 1122, 987, 869 cm^−1^. ^1^H NMR (CDCl_3_, 400 MHz): δ 1.22–1.39 (m, 12H), 1.53–1.58 (m, 4H), 1.89 (m, 2H), 2.71 (t, 2H, *J* = 7.9 Hz), 3.38–3.43 (m, 4H), 3.63 (t, 2H, *J* = 6.6 Hz), 7.21–7.24 (m, 1H), 7.53 (d, 1H, *J* = 7.8 Hz), 8.43–8.45 (m, 2H) ppm. ^13^C NMR (CDCl_3_, 100 MHz): δ 25.71, 26.13, 29.35, 29.45, 29.67, 30.91, 32.76, 62.82, 69.33, 71.01, 123.32, 136.09, 137.36, 147.00, 149.68 ppm. HRMS [M + H]^+^ = 294.2427 (theoretical 294.2433).

12-[3-(pyridin-3-yl)propoxy]dodecan-1-ol **5d**: Yield 73%, yellow oily product: IR (KBr): 𝑣 = 3352, 2924, 2852, 1458, 1433, 1352, 1134, 1114, 985, 858 cm^−1^. ^1^H NMR (CDCl_3_, 400 MHz): δ 1.22–1.39 (m, 16H), 1.53–1.58 (m, 4H), 1.91 (m, 2H), 2.77 (t, 2H, *J* = 7.9 Hz), 3.38–3.44 (m, 4H), 3.62–3.66 (m, 2H), 7.36–7.40 (m, 1H), 7.71 (d, 1H, *J* = 7,8 Hz), 8.49–8.51 (m, 2H) ppm. ^13^C NMR (CDCl_3_, 100 MHz): δ 25.69, 26.13, 29.36, 29.40, 29.51, 29.65, 30.74, 32.69, 62.93, 69.21, 71.09, 124.12, 136.09, 138.48, 138.73, 144.79, 147.33 ppm. HRMS [M + H]^+^ = 322.2742 (theoretical 322.2746).

### 3.3. Drug Samples

The compounds were stored at −20 °C as 10 mg/mL stock solutions in dimethylsulfoxide (DMSO) (Sigma, St. Louis, MO, USA). The compounds were diluted in DMSO and used at a final concentration of 0.01% (*v*/*v*).

#### 3.3.1. Cytotoxicity Assay

The cytotoxicity of the compounds was assessed with the human cell lines, HeLa (cervix adenocarcinoma ATCC# CCl-2), RKO-AS-45-1 (colon carcinoma ATCC# CRL-2579) and WI-26 VA4 (fibroblast ATCC# CCL-95.1) using the MTT (3-(4,5-dimethylthiazol-2-yl)-2,5-diphenyltetrazolium bromide) (Sigma, St. Louis, MO, USA) colorimetric method. Briefly, the cells were plated in 96-well plates (1 × 10^5^ cells/well) and incubated for 24 h at 37 °C in a humidified atmosphere with 5% CO_2_. After 24 h, the wells were washed with culture medium (RPMI +10% inactivated fetal calf serum + 2 mM l-glutamine) and incubated with the compounds at various concentrations (0.05 to 500 μM). After 48 h of incubation, the plates were treated with MTT. The colorimetric measurement was performed at 550 nm using the microplate reader, Spectramax M5e (Molecular Devices, Sunnyvale, CA, USA). Cytotoxicity was scored as the percent reduction in absorbance* versus* untreated control cultures [45]. All experiments were performed in triplicate. The results are expressed as the mean of the IC_50_ (the lethal drug concentration that reduced cell viability by 50%). The IC_50_ values were calculated using OriginPro 8.0 (OriginLab Corporation, Northampton, MA, USA) software. A selectivity index (SI), corresponding to the ratio between the cytotoxic activities of each compound against the HeLa and RKO-AS-45-1 cell lines and the cytotoxicity against WI-26 VA4, was calculated as follows: SI = IC_50_ HeLa or IC_50_ RKO-AS-45-1/IC_50_ WI-26 VA4.

#### 3.3.2. DNA Nick-End Labeling by the TUNEL Method and Immunofluorescence

Apoptotic cell death was measured using the APO-BrdU TUNEL assay kit (Invitrogen, Carlsbad, CA, USA). Briefly, the effect of Compounds **4c** and **5c** and etoposide on DNA fragmentation was determined using Hela and RKO-AS-45-1 cells. The cells were incubated with the compounds for 48 h, as described above, and fixed using a solution of ice-cold 70% ethanol. The cells were then counterstained with 5-bromo-2′-deoxyuridine 5′-triphosphate (BrdUTP) in the presence of terminal deoxynucleotidyl transferase (TdT) and stained with an anti-BrdU monoclonal antibody-PRB-1 Alexa Fluor 488 conjugate, as previously described [14]. The loaded cells were visualized by fluorescence microscopy using a Zeiss Axiovert 200 (Carl Zeiss, Oberkochen, Germany).

#### 3.3.3. Actin Polymerization Detected by Fluorescence Optical Microscopy

RKO-AS-45-1 cells plated in 6-well plates (2 × 10^5^ cells/well) and incubated at the conditions described above were treated with Compound **5c** at 19.1 μM, the same concentration as the IC_50_ determined by the MTT assay. The control with non-treated cells was included in parallel. After 24 h, the cells were rinsed in phosphate-buffered saline (PBS) for 3 min, fixed in paraformaldehyde (#15,812-7, Sigma) at 4% in PBS for 1 h and permeabilized with 0.1% Triton X-100 (#17-1315-01, Plusone, GE Healthcare, Fairfield, CA, USA) in PBS for 10 min. The RKO-AS-45-1 cells were labeled with Phalloidin Alexa Fluor^®^ 488 (#A12379, Molecular Probes, Eugene, OR, USA), and the nuclei were counterstained with 4,6-diaminidino-2-phenylindole (DAPI; Molecular Probes, #D1306) according to the manufacturer’s instructions. Image acquisition was performed using an AxioVert 200 microscope (Carl Zeiss, Oberkochen, Germany).

#### 3.3.4. Cytokinesis-Block Micronucleus Assay

To assess the potential of the synthetic alkaloids to induce DNA damage (chromosomal mutations)* in vitro*, the cytokinesis-block micronucleus assay was performed in the RKO-AS-45-1 cell line. The procedures were developed as described by Fenech [46,47], with adaptations. Briefly, the cells were seeded in 24-well plates (2.5 × 10^5^ cells/well) and maintained at 37 °C in a humid atmosphere with 5% CO_2_. After 24 h, the cells were washed twice with PBS, and the treatments were performed in culture media without serum for three hours. Each treatment was performed in triplicate. The negative control group was treated with PBS, and a positive control group was established with the treatment of the cells with methyl methanesulfonate (MMS—400 μM).

After completing treatments with three concentrations lower than the IC_50_ of Compounds **4c** (4.8, 9.6 and 14.3 μM) and **5c** (5.9, 11.8, and 17.8 μM) diluted in culture media, the cells were washed twice with PBS, trypsinized and centrifuged for 5 min at 1200 rpm. The pellet was then resuspended in chilled hypotonic solution (1% sodium citrate) together with one drop of 1% formaldehyde and carefully homogenized with a Pasteur pipette. This cell suspension was centrifuged for 5 min at 1200 rpm and resuspended in 5 mL of fixative, methanol/acetic acid (3:1 *v*/*v*). Next, the tubes were centrifuged for 5 min, and the supernatant was discarded; the cell suspension was spread onto slides previously cleaned and covered with a film of chilled distilled water.

At the moment of cytogenetic analysis, the slides were stained with DAPI (4′,6-diamidino-2-phenylindole) diluted in phosphate buffer (0.06 M Na_2_HPO_4_ and 0.06 M KH_2_PO_4_, pH 6.8) for 2 min, washed with distilled water and analyzed under a fluorescent microscope (Zeiss, Axioscope) with an excitation filter of 365 nm and a barrier filter of 445/450 nm. Two thousands cells with a well-preserved cytoplasm were analyzed for each treatment in a blind test. Cells containing 1–3 micronuclei were scored. The criterion for the identification of MNs was according to a previous report [48]. A statistical analysis was performed applying ANOVA followed by the Student-Newman-Keuls test.

#### 3.3.5. Frequency of Binucleation (Mitotic Index)

The influence of the synthetic alkaloids on cell division was assessed by calculating the frequency of binucleation in RKO-AS-45-1 cells. The same slides prepared for the micronucleus assay were used, and 300 cells with a well-preserved cytoplasm were counted using fluorescence microscopy, as described above. The frequency of binucleated cells was calculated as the proportion (percentage) of cells undergoing complete cell division after exposure to digoxin and controls [49].

## 4. Conclusions

In conclusion, we demonstrate that novel 3-APA analogs may belong to a promising class of substances with anticancer activity. Among the synthesized compounds, **4c** and **5c** were the most active, mutagenic and induced apoptosis in tumoral human cell lines. Moreover, Compound **5c** altered the cellular actin cytoskeleton of RKO-AS-45-1 cells. Based on these data, Compounds **4c** and **5c** could represent a promising template for developing a new class of anticancer agents, and further investigation of the derived scaffolds is warranted.
